# Two Cases of Aneurism Treated by Ligature without Confinement to Bed—Their Fortunate Termination

**Published:** 1846-02

**Authors:** J. Kirby


*Two cases of Aneurism treated by ligature without confinement to bed—their fortunate termination.* By J. Kirby, Esq.—Since the period in which it was satisfactorily ascertained that ligatures should induce a certain amount of division in the tunics of an artery to secure their successful application, all operators have experienced much anxiety about the degree of force they employ when an artery is being tied, especially in cases of aneurism; but more is esteemed necessary than the due adaptation of force for the purpose of ensuring a fortunate result. Repose is strictly enjoined as an essential to union, and commanded with such authority, that it appears as if the slightest infringement of rule might defeat all previously well-executed proceedings.

The following cases are calculated to abate the great solicitude, which many, in common with myself, have felt upon the latter subject. How far the cautious precept may be neglected or opposed with surprising impunity, the practical reader may judge.

Case 1.—Some years since, assisted by my former pupils, Drs. Mathews and others, I tied the femoral artery at the groin in a man residing in Wexford street. He was young, intemperate, and there was obscure evidence of morbus cordis; of which he died in a few years. He was a thin person. The artery was easily exposed, readily secured, and matters being finished in the usual way, I felt quite satisfied that the case was one of comfortable promise.

The day was Sunday. Monday, and every succeeding day, I saw him morning and evening. The thirteenth day the ligature came away with the dressing, &c,, and the wound was healed.

I was now informed that all my rules, as to rest and quietness, were from the first neglected. Being summoned to a police office a few days before the operation, he left his house the day after it was performed, and walked about a mile to the court, repeating the journey every day for a week, during which his attendance was required. My visits being always at the same hours, he easily carried on the deception, of which one could not hold the least suspicion, as he was always found most comfortably and quietly settled in his bed.

Case II.—I assisted Mr. Mitchell in an operation for popliteal aneurism, performed in the groin. The patient was thirty years old, very plethoric, and very intemperate. Impatient of hospital restraint, he left on the third day, and nothing was heard of him until the fourteenth, when he presented himself in a state of intoxication, with the ligature dangling on his finger, and "wishing his joy to whoever might next require to wear it."—*Dublin Medical Press.*

